# The Optic Nerve and the Accessory Sinuses of the Nose

**Published:** 1907-11-16

**Authors:** 


### Ophthalmology.

### THE OPTIC NERVE AND THE ACCESSORY SINUSES OF THE NOSE.

Everyone who has had experience in diseases of the eye must be quite familiar with many cases of optic neuritis, or atrophy, in which the cause of the condition has been most obscure. Search has been made for other symptoms of the common conditions in which optic neuritis is met with, such as syphilis, cerebral tumour, renal disease, or meningitis, and no ground for such a diagnosis has appeared. The cases may clear up, with restoration of vision and a certain amount of pallor of the disc, or the patients may become and remain totally blind from secondary atrophy, drift away and die in institutions or workhouses, where no post-mortem examination is possible. Little also is known about the real pathology of that interesting affection of one or both eyes known as retrobulbar neuritis. This is a condition of blindness occurring as a rule in young people, women rather than men, affecting one or both eyes, and of very sudden onset. In twenty-four hours vision may diminish from normal to mere perception of light or total blindness, without any apparent fundus change. After a time, usually a few days, vision begins slowly to improve, and coincident with this optic neuritis affects the disc. As the condition is usually monocular, it is evident that the cause of the blindness is situated somewhere between the globe and the optic chiasma, and for this reason the name of retrobulbar neuritis has been selected. It is supposed to be a condition of optic neuritis starting high up in the optic nerve, probably in the optic foramen; a slight amount of swelling and inflammation here will, owing to the rigidity of the bony canal, cause very injurious pressure on the optic nerve, and lead to rapid failure of sight. As the neuritis travels down the nerve, the pressure becomes less and vision improves by the time the neuritis is visible at the optic papilla. The prognosis is usually favourable, and the best remedies are iodide of potassium and tonics containing iron and quinine.

It is now being suggested that some of these cases may be due to inflammation of the various sinuses connected with the nose, which are in close proximity to the optic nerve. The subject has been very long and carefully investigated by Professor Ónodi of Buda Pesth, and is fully discussed in his recent monograph on the subject, "The Optic Nerve and the Accessory Sinuses of the Nose" ("Der Sehnerv und die Nebenhöhlen der Nase," Wien und Leipsig, 1907).

It will be remembered that the optic chiasma lies in the optic groove just in front of the sella turcica and the recess for the pituitary body; the optic nerves run forward through the optic foramina in the lesser wings of the sphenoid into the cavities of the orbits, and it is in the optic foramen that the nerve is only separated by a thin sheet of bone, often no thicker than stout note-paper, from the sphenoidal sinus, or more anteriorly the anterior and posterior ethmoidal cells. In addition to this the frontal sinus on one or both sides may run backwards, and come into close relation with the optic nerve above. The antrum of Highmore in the superior maxilla may also occasionally form a very near relation of the optic nerve below. All these air-containing cavities communicate with the nose, and are not infrequently infected secondarily to affections of the nose; either by the distension of the sinuses by pus, or by periostitis associated with sinus inflammation, the optic nerve may be subjected to sudden and severe pressure in its bony canal. It is quite unnecessary to consider in detail the varying relations of the sphenoidal and ethmoidal sinuses to the optic nerve, though Professor Ónodi, with characteristic thoroughness, describes no less than twelve distinct classes. It is sufficient to remember that these sinuses bear a very close relation to the optic nerve, and that in any case of optic neuritis or atrophy of obscure origin the nose should be very carefully examined for disease.

The literature on the subject is as yet comparatively scanty, but individual cases are by now fairly numerous; the majority of cases, except those that lead to a fatal issue as a result of thrombosis of the cavernous sinus, cannot be proved up to the hilt, unless an operation has been performed for the relief of sinus suppuration. A case such as the following is, however, very suggestive.

A woman aged about thirty-four attended in the eye out-patient department of a general hospital for conical cornea. The vision was examined, and found to be about $\frac{6}{36}$ in each eye, and an operation was advised. On admission to the hospital a few days later it was found that the vision in the right eye had diminished to hand movements, though nothing abnormal could be seen in the fundus oculi. The patient was kept under observation for a few days, but, as the condition remained stationary, she was discharged, and told to attend as an out-patient. Four days after discharge the patient came up suddenly to the hospital, and said that for the last three days she had been suffering from severe headache on the right side, but that yesterday something went "crack" inside her head, and there was a lot of foul-smelling discharge from her nose. Her vision had already much improved, and by the end of a week it had again reached its normal acuity. It seems most likely that this case was one of pressure on the right optic nerve from distension of the sphenoidal or ethmoidal sinus, and that the evacuation of the pus led to speedy relief of the symptoms.

It must be noted that there was no optic neuritis visible, though if the condition had lasted longer the optic disc would doubtless have been affected. As has been mentioned above with reference to many cases, there was no actual proof of any serious empyema in this particular case, though the probability is in favour of this diagnosis, but the very obscurity of the condition makes it imperative that all such cases should be thoroughly examined for nasal disease, and there is no doubt that Professor Ónodi has directed attention in a most striking manner to a class of disease which is of the greatest interest both to the ophthalmic surgeon and the rhinologist.

